# Effects of reintubation on ventilator associated nosocomial pneumonias incidence in ICU patients with versus without selective digestive decontamination

**DOI:** 10.1186/2197-425X-3-S1-A704

**Published:** 2015-10-01

**Authors:** AM Marrero-Rodríguez, ER Argandoña-Primicia, ME Gallardo-Santos, P Juárez-San Juan, CF Lübbe-Vazquez, JJ Díaz Díaz, P Saavedra, S Ruiz-Santana

**Affiliations:** University Hospital of Gran Canaria Dr. Negrín, Intensive Care Unit, Las Palmas de Gran Canaria, Spain; Las Palmas of Gran Canaria University, Mathematics and Informatics Department, Las Palmas de Gran Canaria, Spain

## Introduction

Reintubation has been shown, among many others, to be an independent risk factor for development of ventilator-associated pneumonia_1_ (VAP) which is a major cause of morbidity and mortality in ICUs. Selective Digestive Decontamination (SDD) is a tool that prevent infections in critically ill patients particularly VAP, that has been used mainly in Holland and in Spain. Despite of the evidence its use remains controversial.

## Objectives

To assess the VAP incidence after reintubation in patients admitted to a polyvalent ICU before and after starting a SDD program.

## Methods

A retrospective study was conducted in a 30-bed-medical-surgical ICU. We compared all patients admitted to ICU who required reintubation from October 2009 to October 2011 (non-SDD group) to those patients admitted to the ICU from October 2011 to October 2013, after starting a SDD program (SDD group) prospectively collected data. All patients that were expected to require tracheal intubation for longer than 48 hours were given SDD (SDD study group). Nosocomial infections were diagnosed by CDC criteria. In both groups, categorical variables were summarized as frequencies and percentages and the continuous ones as medians and interquartile ranges (IQR). The percentages were compared using the test of chi-square test or Fisher exact test and medians with the Wilcoxon test for independent samples. For VAP the incidences per 1000 days of mechanical ventilation were obtained. These rates were compared using the Poisson model. Statistical significance was set at *p* ≤ 0.05.

## Results

**Figure 1:**
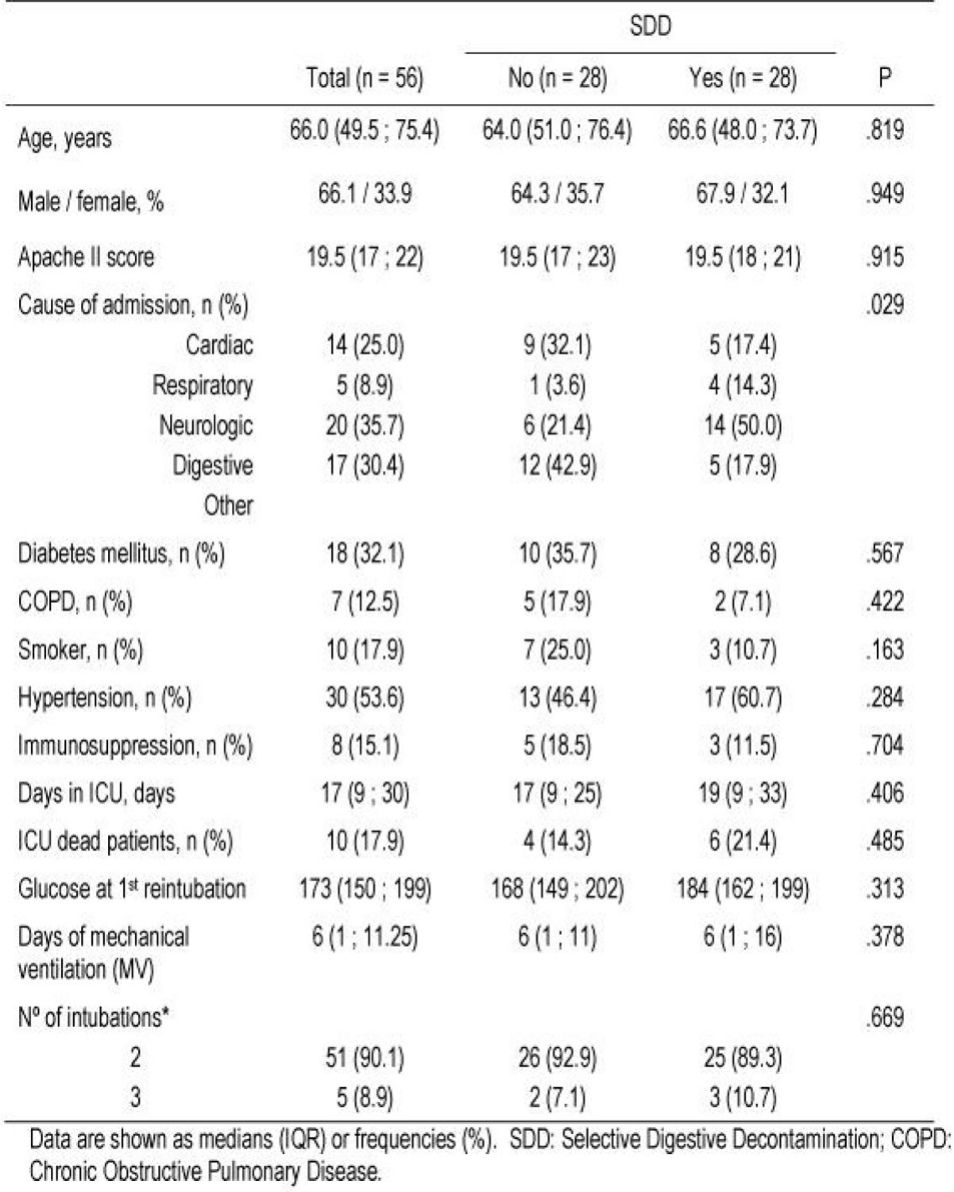
Figure 1

**Figure 2:**
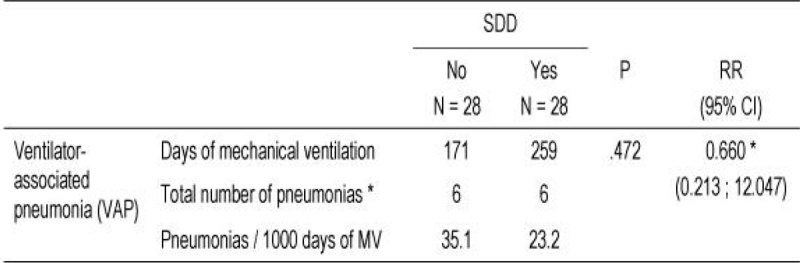
Figure 2

Results are shown in Figures 1, and 2. There was a non-significant reduction of VAP per 1000 days of mechanical ventilation (23.2 vs. 35.1) in patients that were reintubated in the SDD period (n=28) compared to those in the non-SDD period (n=28). The risk reduction observed was 34%.

## Conclusions

There is a notorius reduction, towards significance in the incidence of VAP per 1000 days of mechanical ventilation in patients reintubated on SDD compared to those reintubated in the non-SDD period.
